# How Private Health Insurance Shapes Perceptions of Public Healthcare in Sweden

**DOI:** 10.1002/hpm.3941

**Published:** 2025-04-23

**Authors:** Linn Kullberg, Paula Blomqvist, Ulrika Winblad

**Affiliations:** ^1^ Department of Public Health and Caring Sciences Uppsala University Uppsala Sweden; ^2^ Department of Government Uppsala University Uppsala Sweden

**Keywords:** expectations, healthcare systems, private health insurance, public healthcare, satisfaction, Sweden, voluntary health insurance

## Abstract

The increasing prevalence of private health insurance (PHI) in tax‐funded healthcare systems challenges the principles of equity and universalism. A significant proportion of PHI holders in such systems receive their insurance as an employment benefit, granting them access to privately funded healthcare alongside the publicly funded system. This dual access raises critical questions about how individuals navigate between the two sectors and how their experiences shape their perceptions of public healthcare. The aim of this study is to explore how the use of PHI‐funded healthcare services influences perceptions of and satisfaction with the public healthcare system. Specifically, we examine when PHI holders choose privately funded care over public services, how they perceive the two sectors, and whether they would purchase PHI independently if it were not offered as an employment benefit. An interview study was conducted in 2022 with 19 individuals in Sweden who receive PHI as an employment benefit. Using thematic analysis, the findings reveal a preference for privately funded services due to faster access and higher service quality. However, the medical quality of specialised care in the public sector is still regarded as high. PHI is perceived as providing a sense of security through prompt care, but few respondents expressed a willingness to purchase it privately, suggesting it is seen more as a convenience than a necessity. These findings highlight the role of PHI in shaping expectations and satisfaction within tax‐funded healthcare systems, offering insights into its potential impact on public trust and support of universal healthcare.

**Summary**

- Explores how PHI impacts perceptions of public healthcare in tax‐funded systems.
- PHI holders prefer private care for faster access and better perceived quality.
- Few PHI holders would buy PHI privately, finding it too costly for its value.
- Access to PHI may raise expectations and demands on public healthcare.

## 1 Introduction

International evidence indicates that in countries with a substantial private healthcare sector, public healthcare is often perceived as inferior [1, 2]. This perception prompts those who can afford it to opt for private options, leaving those with lower incomes to rely on public services [2, 3, 4, 5]. Such dynamics, which creates two‐track healthcare systems, pose a particular challenge to the universal, tax‐based, healthcare systems found, for instance, on the British Isles and in the Nordic countries. This type of system is based on the idea that all citizens should have equitable access to healthcare, regardless of income or employment status [6, 7, 8]. Even these systems have, however, seen a growth in private health insurance (PHI) over the past decades [9, 10]. Real and perceived gaps and inflexibilities in public systems, along with policy incentives such as tax exemptions for employer‐funded PHI, are suggested drivers of this development [9]. In tax‐funded healthcare systems, PHI is often *duplicating*, meaning it covers healthcare services that are also provided within the public healthcare sector. This means that PHI holders have access to these services in both public and private settings, allowing them to choose where to receive them [11]. This double access, and opportunity to compare health services in the public sector with those offered through PHI, has raised concerns that the private sector will be seen as more attractive and, in the end, lead to a decline in support for the public healthcare system. If this happens, the solidaristic basis of the public healthcare system is threatened, increasing the risk that it will be perceived as a second‐rate option, even within universal, tax‐funded healthcare systems. So far however, there is not much knowledge about how PHI holders in such systems navigate between the two healthcare sectors they have access to (public and private) or how their perceptions of and satisfaction with the public system is affected by having a PHI.

Sweden represents a case of a tax‐based healthcare system where PHI has become significantly more common in the past decades. In 2022, nearly 14% of the working population had access to PHI, in most cases obtained through employment [10]. Typically, such insurance provides faster access to primary care and certain outpatient specialist care. At the same time, the modern Swedish healthcare system has been constructed with the explicit goal that access to healthcare services should be equal for all citizens, regardless of income or occupation. Part of the strategy behind this universalistic objective was to ensure that the public healthcare system provided services of such high quality that also the better‐off, who could afford to turn to the private market, would choose to remain in the public healthcare system [12, 13].

In this context, the increased uptake of PHI represents a challenge in several ways. First, the simple fact that some citizens, typically those with higher incomes, can use PHI to secure quicker access to healthcare in a privately funded sector undermines the principle of equal access. Second, and perhaps even more problematic, some perceive a risk that the use of PHI, which in most cases only covers a restricted range of health services, predominantly in the outpatient sector, will potentially raise overall expectations for healthcare delivery, creating a disparity between PHI users' expectations and what the universal healthcare system can offer [2]. This, in turn, may create a snowball effect, leading to declining trust in the public system and a growing demand for more services to be covered by PHI. In this case, a two‐tier system will develop also in a tax‐based system like the Swedish and the goal of equity will be diminished. This will only happen, however, if PHI holders come to see privately funded health services that they get access to as superior to those provided through the public system, and that they or their employers are willing to purchase it.

In this light, the aim of this study is to explore how the experience of accessing and utilising PHI‐funded healthcare services influences individuals' perceptions of, and satisfaction with, a publicly funded healthcare system.

1. When do PHI holders choose privately funded healthcare over public healthcare?
2. How do PHI holders perceive the public healthcare system in comparison with the private healthcare sector?
3. Would individuals with employer‐provided PHI be willing to purchase it independently using their own funds?

In order to answer these questions, a qualitative approach based on semi‐structured interviews was chosen. This approach contributes to a deeper understanding of how PHI holders combine publicly funded healthcare services with PHI‐funded alternatives and how they perceive public healthcare in the light of the experience of PHI‐funded healthcare.

The findings suggest that PHI holders prefer privately funded services over publicly funded healthcare services when given a choice. Particularly in terms of access and within primary care and outpatient specialist care, the private sector is perceived to outperform the public sector. Overall, the results suggest that access to PHI‐funded healthcare provides a sense of security through the knowledge that health needs will be addressed promptly. However, only a few respondents receiving PHI through their employment said that they would be willing to purchase PHI with private means, suggesting they still see this a relatively marginal benefit.

The findings in this study offers valuable insights into the influence of employer‐funded PHI on healthcare choices and perceptions within systems where publicly financed healthcare is predominant. By examining the attitudes of insurance holders in a universalistic healthcare system like the Swedish, it fills an important gap in international research, providing an enhanced understanding of the role of PHI in this type of healthcare system.

The paper is organised as follows: First, a brief introduction to PHI in tax‐funded healthcare systems is presented, followed by a review of the literature on PHI holders' perceptions of publicly and privately funded healthcare. This is succeeded by an introduction to the case study setting and a description of the methods used. The fourth section of the paper presents the empirical results, followed by a concluding section where these results are discussed.

## 2 Private Health Insurance in Tax‐Funded Healthcare Systems

PHI exists in most OECD countries, although its role in healthcare systems varies. In tax‐funded healthcare systems, PHI is most often voluntary. Citizens are not able to opt out of the system, and they continue to contribute to the public healthcare system by paying income tax. PHI offers a way to complement and duplicate public healthcare services by providing access to similar services in the private sector—often with shorter waiting times—and, in some cases, coverage for high out‐of‐pocket expenditures within the public system. In this way, PHI holders benefit from a form of dual coverage—through the public healthcare system and their PHI. PHI is most commonly provided by employers as an employment benefit. It can also be offered as a group policy through professional organisations and unions; however, in these cases, the cost is typically borne by the individual policyholder. A third category includes individuals who purchase private policies on their own [14]. The size and importance of the private insurance market varies between countries. In Italy, about 24% of the population has PHI [15]. In Spain, the corresponding number is 15% and 10% of the population in the UK have PHI [16]. There is also variation among the Nordic countries: about 12% hold PHI in Norway, 24% in Finland and 37% in Denmark [17]. In Sweden, the market for PHI has grown significantly during the last decades. In 1990, only 23,000 individuals held PHI, a number that had multiplied to 760,000 persons by the end of 2022. This corresponds to about 7% of the population, the majority of whom receive it as a benefit from their employer [18].

### 2.1 Demand for Private Health Insurance

Numerous studies have explored the factors driving demand for PHI, with evidence highlighting the significant influence of socioeconomic status. Individuals with higher incomes, higher educational level, and younger age are more likely to obtain PHI [4, 5, 19, 20, 21, 22, 23].

Another factor that may drive demand for PHI is the performance of the public healthcare system, particularly in terms of low satisfaction and perceived service quality. A study conducted in Spain found that demand for PHI was influenced by a perceived quality gap between publicly funded healthcare and that funded by PHI [1]. Another study revealed that PHI holders were more likely to believe that the public healthcare system required reformation [5]. In a third study from Spain it was found that satisfaction with the public healthcare system was lower among PHI holders compare with people without a PHI [1]. However, it remains unclear whether holding PHI leads to reduced satisfaction with public healthcare, or if dissatisfaction motivates individuals to purchase PHI.

In a study from England, with the aim to discover which groups of people chose private care rather than using the NHS and why they did so, it was found that the most common reason given among PHI holders to use private healthcare was to avoid waiting times in the public healthcare sector [24]. A factor that consistently have been recognised as a significant driver of PHI uptake [4, 25]. Previous negative experiences with the public system were also cited as a contributing factor [24]. Other factors influencing demand for PHI include high co‐payments within the public healthcare system [4, 21], concerns over poor cleanliness in public hospitals [21], and negative media coverage of the public healthcare system [24, 26]. Also in Portugal, the NHS has relatively high out‐of‐pocket expenditures, which is considered a driver of PHI uptake [23].

In addition to dissatisfaction with the public healthcare system, previous research has identified various ‘pull' factors that make PHI‐funded healthcare appear more appealing than the public sector. These factors include the ability to choose physicians and appointment times more freely, perceptions of higher care quality in the private healthcare sector, and greater trust in privately funded healthcare providers [4, 24]. Another factor motivating PHI adoption is access to dental care, which is not covered by the NHS in Portugal [23]. Evidence from Italy further highlights how structural factors influence PHI utilisation. Individuals in regions with better healthcare infrastructure and service availability are more likely to use PHI, suggesting that access to services plays a role in shaping demand for PHI [15]. A Finnish study found that PHI holders expressed a desire for access to the highest possible quality of care, even for minor healthcare needs, which they believed was more attainable in the privately funded sector [2].

#### 2.1.1 Perceptions of, and Satisfaction With Public and Private Healthcare

While there are numerous studies focussing on the demand for PHI, only a few studies have examined perceptions of and satisfaction with the public healthcare system among PHI holders. Patient satisfaction is widely recognised as a complex, multidimensional concept that encompasses various aspects of the healthcare experience as perceived by patients. Several reviews have sought to identify key determinants of patient satisfaction [27, 28, 29]. These determinants can be categorised into various aspects, such as healthcare outcomes, access, caring, communication, and facilities [29]. Among these, caring—or “interpersonal care”—emerges as one of the most significant determinants of patient satisfaction [27, 28]. This category includes various facets of care, such as active listening, participation, offering comfort, friendliness, and demonstrating empathy and sympathy [27]. A closely related dimension is communication, which involves both the quality of information provided and the communication skills of healthcare providers [29].

Despite the complexity of the concept, patient satisfaction is often used in both national and international surveys, as a measure of how satisfied the general population is with the healthcare system at large. The aim of these studies has often been to compare different healthcare systems and to identify determinants for public satisfaction with the healthcare system. Studies have found lower levels of satisfaction in healthcare systems with fewer general practitioners [30], as well as in system with lower levels of expenditures on healthcare [30, 31], and in systems with high co‐payments [30]. Implying that the level of public resources spent on healthcare is important for how satisfied the population is with the healthcare system. Popic and Moise (2025) find that healthcare privatisation initially leads to more positive evaluations of the health system, particularly when reforms expand choice. However, over time, evaluations decline, especially when privatisation introduces out‐of‐pocket costs and reduces access to care [32].

In Australia, where approximately half the population holds PHI, the private healthcare sector is larger than in most European systems and offers access to both advanced and acute medical services, forming a full two‐tier system. In this context, studies suggest that lack of trust in the public system is a key factor influencing PHI uptake [33, 34]. Concerns such as underfunding, long waiting times, and understaffing in the public sector were commonly cited, alongside negative media coverage of public healthcare. Conversely, personal experience with the public system tended to enhance trust in its services [33].

The literature reviewed in this section suggests that perceptions of inferior quality in publicly funded healthcare can be a driver in the decision to purchase PHI. In addition, it seems like satisfaction with the public healthcare system could be reduced when compared with experiences in the privately funded health sector. However, these studies have primarily been conducted in contexts where the private sector is significantly larger than in Sweden. Additionally, respondents in the studies have usually bought the PHI with private means, which may increase their propensity to highlight its benefits.

In this study, we focus on individuals who receive PHI as an employment benefit, a group of particular interest for several reasons. First, unlike those who actively choose to purchase PHI and pay the full premium privately, the individuals in this study receive it as part of their employment package and only contribute a smaller portion through benefit taxation. As such, they are less likely to feel the need to justify the superiority of privately funded healthcare services. Secondly, this group is noteworthy because it predominantly consists of middle‐class individuals [35]. This demographic typically acts as net contributors to the public healthcare system and has the financial capacity to access private healthcare independently [36, 37]. Over time, positive experiences with PHI‐funded services could lead this group to favour private healthcare over public options, potentially eroding middle‐class support for the public healthcare system.

## 3 Case Study Context: The Swedish Healthcare System

Sweden has a tax‐based universal healthcare system of a national health service (NHS) type, where all citizens and permanent residents have access to a broad range of healthcare services at low cost. The Swedish system is decentralised to 21 Regions, where regional authorities are responsible for the provision and funding of healthcare services, which mainly comes from regional income taxation [38]. Solidarity in financing and equity in access are central principles in the system. The Health and Medical Services Act (SFS 2017:30) mandates that healthcare be allocated based on medical need. However, it does not grant patients the right to specific treatments, only outlining what the system should provide [39].

The provision of healthcare services in Sweden have long been mainly public [13, 40], with the majority of hospitals and outpatient specialist care units owned and managed by regional authorities. However, market‐oriented reforms introduced in the early 1990s have led to an increased presence of private providers within the system, particularly in primary care. Private providers may be contracted by regional authorities to deliver publicly funded healthcare services or operate under private insurance schemes [38]. Thus, in the Swedish context, the public healthcare system entails both privately and publicly operated clinics. By 2018, over 40% of all publicly funded primary care clinics were privately operated, and approximately 20% of outpatient specialist care visits occurred in privately operated clinics [38]. Notably, it is not permissible to opt out of the tax‐funded Swedish healthcare system in favour of a fully private alternative.

The Swedish healthcare system is often highly ranked in international comparisons of medical quality but tends to score lower on aspects such as patient involvement and information provision. Long waiting times for certain treatments also remain a persistent challenge [38]. While there is no formal gatekeeping system, access to secondary care specialists frequently requires a referral from a general practitioner [38]. One distinction between private and publicly funded patients is the significantly shorter waiting times offered by PHI. Patients with PHI are typically guaranteed treatment or surgery within two to three weeks, whereas the public system has a maximum waiting time guarantee of 90 days [41, 42].

### 3.1 Private Health Insurance in Sweden

The expansion of PHI is a relatively recent development in Sweden. Predominantly duplicating, Swedish PHI covers services already provided by the public healthcare system. Its primary purpose is to offer faster access to private healthcare services rather than to cover out‐of‐pocket expenses for public healthcare, which remain relatively low due to the national high‐cost protection scheme. Most PHI holders, about 60%, receive their policy as a fringe benefit from their employer. The second largest group, approximately 30% has a group policy offered through a professional organisation or union. However, in contrast to employer‐funded PHI, these policyholders pay the premiums themselves. Only about 10% of all PHI holders in Sweden purchase an individual policy privately. The cost of PHI varies depending on how it is obtained. Individually purchased policies costs between €200 and €600 per year. For employer‐funded policies, most of the premium is covered by the employer, but employees pay income tax on about 60% of its value, typically amounting to €26–43 per month [42]. All in all, about 14% of the working population in Sweden had PHI in 2022, corresponding to about 7% of the entire population [10]. Although a substantial part of the population has access to PHI, the funding of such insurance makes up less than 1% of Sweden's total healthcare expenditure [43]. Previous research indicates that PHI holders are typically younger, have higher incomes, and are more likely to be employed in sectors such as financial services, legal and economic consultancy, and construction, compared to non‐PHI holders [44].

Most PHI plans cover elective specialist care, rehabilitation, and psychological sessions, with some plans also including less severe cancer treatments. While the insurance is predominantly used for outpatient specialist care such as orthopaedics, dermatology, and physiotherapy, it may also cover certain inpatient treatments. However, it excludes many services, including acute medicine, intensive care, highly specialised care, fertility treatments, and reproductive health services. Pre‐existing conditions are also typically excluded from coverage [35, 45].

To access PHI services, the insured individual contacts a care coordinator at the insurance company—often a registered nurse—who conducts an initial assessment and assists in arranging an appointment at one of the insurer's affiliated clinics. Although PHI holders and private healthcare clinics are present across Sweden, the majority of these clinics are concentrated in the Stockholm area, the nation's capital.

## 4 Methods

A qualitative approach based on semi‐structured interviews was used in this study [46, 47]. Since there is no public register of individuals with an employer‐funded PHI in Sweden, sampling and recruitment were based on personal contacts and a snowball technique. Purposeful sampling was applied with the aim of reaching a homogeneous sample of typical PHI holders [48].^(p243)^ By limiting the geographical recruitment to Stockholm, potential variation in accessibility to private clinics across the country, which could potentially bias the result, was minimised. All respondents had experience of both public healthcare and PHI funded services. All respondents but one had a university degree and most were employed in the sectors of finance, engineering, and IT. Eight women and 12 men between the ages of 33 and 59 were interviewed.

In total, 20 interviews were conducted by the first author between February and October 2022. However, during the analysis, one interview was removed since it was found that the respondent did not fulfil all the criteria for inclusion. The interviews were conducted on the video‐meeting programme Zoom and lasted between 26 and 83 min. All interviews were carried out in Swedish. Selected quotes were translated into English by the authors. All respondents signed an agreement to participate.

During the interviews, respondents were asked to describe their experiences of using both PHI funded services and public healthcare, and to reflect on perceived differences. Participants were also asked about the circumstances and reasons behind their choice between PHI and publicly funded healthcare services, as well as whether they would consider purchasing PHI independently if it were not offered as an employment benefit.

In order to investigate how PHI holders experienced the use of PHI funded healthcare services and how they perceive potential differences between publicly and PHI funded healthcare, thematic analysis was used [49, 50]. Thematic analysis is a method for organising qualitative data by identifying, analysing and reporting central themes in relation to the research topic. Braun and Clark provide a guide of the different phases of thematic analysis. In the first phase of the analysis, the transcripts were read for familiarisation, followed by a phase of an initial inductive coding. In this phase, selective coding was applied, where only text segments related to the research questions were coded. In the third phase, the identified codes were organised into potential themes. In the work of sorting codes into themes, sketches of so‐called thematic maps were made to visualise the relationship between individual codes and how they could be grouped into larger themes. The process of sorting codes and building themes involved going back and forth between the data and the thematic maps until the themes were settled. In the fourth phase, after all interviews had been analysed, the material was all reviewed again to consider the validity of the individual themes in relation to the data. In the fifth phase, the themes were defined and named.

The software programme NVivo 1.5 was used for the analysis. Ethical approval for this study (Dnr: 2020–01982) was granted by the National Board of Ethical Review.

## 5 Results

### 5.1 How do PHI Holders Choose Between the Private and Public Sectors?

The first research question explored in this paper concerns how PHI holders utilise their insurance. Specifically, in what circumstances do they use their PHI to access health services, and when to they turn to the public healthcare system? Analysis of the interview responses reveal a clear preference among respondents for using their PHI whenever feasible. The primary reason cited was the perception that PHI funded services are superior, particularly in terms of service quality and accessibility. Respondents also reported finding it easier to schedule an appointment through their PHI, with several highlighting the booking system as a significant factor influencing their choice of PHI over the public healthcare system. Another key reason was the ability to access specialist care directly without having requiring a referral from a GP, a step that is often needed within the public system.

Why should I waste time with this doctor [publicly funded GP] when I know it's a specialist I need? I know I need to get to a dermatologist. Uh, so why should I take, like, an hour of this doctor's time?

(Interview 16)

A few respondents stated that they used their insurance to obtain a second opinion. Some described making case‐by‐case assessments, weighing whether it was worth paying the SEK 500–700 deductible to activate their insurance. For example, they sometimes used the insurance to address concerns about a mole or other health issues. Others mentioned turning to the private sector for issues they wanted resolved quickly but which were not prioritised within the public system. As one respondent explained:

But if it's something that I know will take a two‐month waiting period, or three or four, or may be completely unprioritised [in the public sector] but which you still need help with before it becomes a prioritised thing—like, is it skin cancer or not? —then it is perfect to have the insurance

(Interview 2)

The reasons for turning to the public healthcare sector varied among respondents. A few respondents indicated that their first choice was public healthcare, either due to ideological reasons or out of habit. Some reported initially seeking care at public clinics but ultimately switching to their PHI due to excessively long waiting times. Others stated that their choice of sector depended on the nature of their medical needs. For emergency care or severe illness, the public system was regarded as the only option. Additionally, since pre‐existing conditions are excluded from PHI coverage, long‐term conditions predating their insurance had to be managed within the public system.

### 5.2 How do PHI Holders Perceive the Public Healthcare System?

To address the second research question, respondents were asked to elaborate on their perceptions of differences between PHI‐funded and public healthcare services. Their responses were categorised into three main themes: *access, service quality,* and *medical quality*, with each theme further divided into sub‐themes to capture the nuances of their experiences and perceptions.

#### 5.2.1 Access

Access was identified as the primary difference between PHI‐funded and public services, according to respondents. The discussion of access revolved around two sub‐themes.

##### 5.2.1.1 Flexibility and Convenience

Many respondents emphasised the efficiency and ease of accessing care through their PHI. They particularly valued the flexibility to choose both the time and location of their appointments. One participant highlighted the convenience of selecting a provider based on proximity and detailed information:

Make a call [to the insurance company] and get help immediately. And get, like, different options on where I can go that suits my schedule and my day […] I could click on a map and, like: ‘Thomas’—This is Thomas, his background, experience etc.’ And I, like, perfect! [He was located] 300 m from my workplace.

(Interview 18)

In addition, several respondents appreciated the assistance provided by their PHI in booking appointments. The assurance of timely communication and follow‐up was seen as particularly valuable:

[You get] confirmation that a registered nurse will contact you within 24 hours. That peace you get by knowing that someone will take care of you within 24 hours…

(Interview 19)

##### 5.2.1.2 Waiting Times and Barriers to Access

A common theme was the significantly shorter waiting times in the PHI‐funded sector. Many respondents reported securing appointments on the same day or the following day, with PHI‐funded providers often offering swift scheduling for procedures such as MRIs or surgeries.

By contrast, public healthcare was frequently described as less accessible. Visits to emergency rooms were associated with expectations of long waiting times; in addition, appointments at primary care centres often required persistent effort. Respondents particularly highlighted extended lead times for further examinations and treatments within the public system. Several participants shared how seeking care in the public sector could feel like a struggle, requiring them to actively "fight” for adequate assistance. One respondent expressed the belief that one had to exaggerate symptoms to secure a first appointment in the public sector.

Maybe you have to exaggerate a little, like your symptoms. Or Google a little and appear a bit worse than you are to get that first appointment.

(Interview 5)

Another respondent described how he had used strategic word choices, such as; chest pain, to be prioritised. A third remarked, with a laugh, that “you should definitely not wear make‐up,” implying that appearing tired and unwell increased the likelihood of being taken seriously.

While most respondents emphasised access speed as the key difference between the two sectors, several stated that once access was achieved, the medical quality was comparable:

Finding your way in and finding the person you need to talk to is very much quicker in the private sector. But once you find the right one [physician], the difference […] is marginal. It's just that it goes much faster through the private sector.

(Interview 8)

Not all respondents reported negative experiences with the public healthcare system. A few described their listed public primary care centres as functioning well and noted no difficulty in securing appointments. Some even reported shorter waiting times in the public sector compared to privately funded clinics. Additionally, several respondents expressed confidence that in cases of acute illness, the public system would provide timely access to necessary care.

#### 5.2.2 Service Quality

Service quality emerged as a significant theme in the interviews, with three sub‐themes identified during the analysis. While many respondents expressed a preference for PHI‐funded healthcare across these dimensions, some noted similarities between the two sectors or attributed differences to individual practitioners rather than systemic factors.

##### 5.2.2.1 Perceptions of Atmosphere and Culture

A majority of respondents highlighted distinctions in the atmosphere and culture between the two sectors. Privately funded clinics were frequently described as calmer, more peaceful, and welcoming environments. Respondents appreciated the attention to detail, such as comfortable waiting rooms, coffee machines, and the personalised approach of staff, which made them feel more like valued customers than patients. As one participant explained:

I feel more like a VIP when entering [a privately funded clinic; […] they say, like, ‘nice to have you here’ […] It’s not like [public clinics where] you get a queue slip with an instruction to wait on a plastic couch.

(Interview 15)

Some respondents also noted experiences of differential treatment in terms of interpersonal interactions. For instance, one participant described overhearing a receptionist reminding a treating physician that he was a PHI‐funded patient:

They definitely treated me differently. What other customers they have, I don’t know.

(Interview 7)

##### 5.2.2.2 Responsiveness to Patient Needs

Another key dimension was the extent to which respondents felt their needs were taken seriously. While some believed that the individual encounter depended more on the personality of the physician than the sector, most felt that PHI‐funded providers excelled in this regard. Respondents emphasised that private healthcare staff often took the time to listen carefully, address concerns thoroughly, and conduct comprehensive examinations.

In contrast, public providers were often perceived as less responsive, characterised by “tired” environments where staff appeared stressed and hurried. Several respondents reported feeling like a burden in these settings—treated as someone to be managed swiftly, regardless of whether their concerns were fully addressed. One respondent noted:

It can be quite rapid and humiliating… You have to convince them that it is a real problem, not just something that you experience, a little unpleasant feeling.

(Interview 5)

This perception contributed to an expectation among some respondents that public clinics would provide only minimal treatment, such as being advised to take aspirin and return if their condition worsened.

##### 5.2.2.3 Continuity and Transparency

Continuity of care and transparency were also highlighted as key dimensions of service quality where the private sector was seen as superior. Many respondents expressed frustration with a perceived lack of continuity within the public system, where they often encountered different physicians and struggled to identify who was responsible for their care. In contrast, PHI‐funded healthcare was praised for ensuring continuity, with one caregiver overseeing the patient's journey from initial contact to recovery. This consistency provided respondents with a sense of security and trust.

#### 5.2.3 Medical Quality

Respondents varied in their perceptions of medical quality and whether it differed between public and PHI‐funded providers. Some emphasised that they lacked the expertise to assess differences in medical quality directly. However, some respondents considered PHI‐funded healthcare services to be of higher medical quality. These views were often linked to specific negative experiences with public healthcare, such as inadequate nursing care in emergency rooms, incomplete diagnostics, or insufficient treatment at public clinics. A few respondents expressed concern that long waiting times in the public sector could exacerbate medical conditions, potentially leading to poorer outcomes.

##### 5.2.3.1 Recruitment and Specialisation

Some respondents believed that PHI‐funded clinics had an advantage in recruiting experienced staff, suggesting that physicians in public primary care centres were often early in their careers, which might affect medical quality. Several respondents emphasised the benefit of directly accessing specialists through PHI‐funded healthcare without needing a referral from a general practitioner. Public primary care centres and general practitioners were often viewed with scepticism, with some respondents questioning their competence. As one participant noted:

Instead of seeing a general practitioner, you get to see someone who has spent their whole life looking at a weird‐sounding knee. And that means that there is a sense of security, that you feel that you are well taken care of right from the start.

(Interview 3)

##### 5.2.3.2 Thoroughness and Diagnostic Practices

Several respondents highlighted the perception that PHI‐funded clinics provided “more” healthcare, particularly in terms of testing, investigations, and follow‐ups. Private providers were often viewed as more thorough, offering services such as MRIs, ultrasounds, and regular check‐ups that were less common in the public system. One respondent shared their experience:

I've had [private] specialists who really check my whole body. Even if it's sleep problems, they have, like, taken blood samples once every six months to check that metabolism and everything like that is ok… So, it feels like they are very thorough.

(Interview 17)

##### 5.2.3.3 Concerns About Over‐Treatment

Despite the generally positive perceptions of private healthcare, a couple of respondents expressed concerns about the incentive structure in the PHI‐funded system, suggesting that it might sometimes lead to unnecessary interventions. One participant reflected on the experience of being quickly scheduled for surgery and considered whether physiotherapy might have been a more appropriate option.

#### 5.2.4 Shifted Expectation

Taken together, the respondents presented a relatively consistent picture: accessing PHI‐funded healthcare services was perceived as significantly quicker and easier compared to the publicly funded services. While most respondents acknowledged that they lacked the expertise to evaluate differences in medical quality, the majority expressed high satisfaction with the care they received at PHI‐funded clinics. In contrast, the public sector was often criticised for issues related to access and service quality, particularly within primary care. However, specialised and highly specialised care within the public system was widely appreciated and regarded as trustworthy.

Participants' experiences with private healthcare had a notable impact on their satisfaction with public healthcare. The ability to compare the two systems often highlighted the benefits of PHI‐funded services, with some respondents noting that this comparison raised their expectations of public healthcare. As one participant remarked,

I didn't have so many demands before […] whereas now, when you get super‐help every time [at the private clinics] you get used to it and you place higher demands on public healthcare, which they probably can't live up to.

(Interview 3)

This shift in expectations underscores how exposure to private healthcare may reshape perceptions of public healthcare.

### 5.3 Would Individuals With Employer‐Funded PHI be Willing to Purchase It Independently?

The third research question explored whether employer‐funded PHI holders would be willing to purchase insurance independently if it were not provided by their employer. Most respondents viewed their PHI as only a fringe benefit, and only a few indicated that they would purchase it using their own funds if it were no longer offered by their employer. However, some noted that the importance of this benefit had increased with age and the emergence of new medical concerns. As one respondent explained:

I am almost 60, and then things start to happen. So, the value [of it] is greater for me maybe than for my colleagues that are 25 and 35 and 45 […] I think the value exceeds the cost.

(Interview 16)

A few respondents had purchased additional insurance for family members, while one participant, who had changed jobs to an employer that did not provide PHI, chose to continue paying for the insurance privately to retain it. Despite these examples, most respondents stated that, although they valued the benefit, they would likely not purchase private insurance independently. Some also mentioned that they found the cost of purchasing PHI individually to be prohibitively expensive.

## 6 Discussion and Conclusion

Based on 19 interviews, this study explored how experiences with PHI‐funded healthcare services shape individuals' perceptions of and satisfaction with Sweden's public healthcare system, with a focus on utilisation patterns, comparisons between public and private healthcare, and the willingness to purchase PHI privately. While the sample size is appropriate for a qualitative study, it does not indicate how widely shared these attitudes are in the broader population. Nevertheless, the findings highlight key themes and perceptions that may be relevant beyond this sample.

The results indicate a preference for PHI‐funded services, primarily due to faster access, a result consistent with previous research [4, 25]. Booking appointments through PHI was described as simple and efficient, in contrast to the more time‐consuming process associated with public healthcare. While the quality of specialist and hospital care in the public sector was generally trusted and regarded as good, primary care and long waiting times for specialist and hospital services were key areas of concern. Respondents also felt that their medical needs were taken more seriously by PHI‐funded providers. Overall, the results suggest that access to PHI fosters a sense of security by ensuring timely attention to health concerns, though it also appears to shift expectations, leading some respondents to place higher demands on public healthcare that it may struggle to meet.

Universalism and equity are foundational principles of the Swedish healthcare system. However, the expansion of PHI as an alternative to the public system suggests a shift away from universalism. This is because access to PHI‐funded healthcare is increasingly determined by ability to pay or employment sector, rather than medical need. A key component of universalism in the Nordic context is comprehensive usage, meaning that the majority of citizens rely on the public system rather than private markets. This principle is linked to the idea that maintaining middle‐class support is crucial for sustaining public backing of the welfare system [36, 37]. To retain this support, it is seen as essential to deliver public services of such high quality that middle‐class citizens find them satisfactory and do not turn to private providers for equivalent services. If a significant proportion of the population acquires PHI, there is a risk of diminishing willingness to pay taxes for the public healthcare system. This could result in fewer advocates for investment and quality improvements in public healthcare, potentially leading to a decline in the quality of public services—not only relative to the private sector but in absolute terms. A similar development was observed in the UK, where regions with a high density of PHI holders appeared to invest less in reducing waiting times in the public healthcare sector [51].

What this study illustrates is that there is a lack of satisfaction with the public system regarding access and service quality within primary care and lengthy waiting times in specialist ‐ and hospital care. Against this backdrop, the universalistic goal of the Swedish healthcare system—to deliver services of such high quality that even the middle classes prefer them over private alternatives—has, to some extent, fallen short. In this regard, the growth of the PHI market poses a challenge to the public healthcare system. On the other hand, the findings of the study also suggest that the majority of those obtaining a PHI as an employment benefit today would not be willing to pay the full cost for it themselves. This suggests that the perceived threat of PHI to support for the public healthcare system may be overstated. Future research would benefit from survey‐based studies to assess how widespread these expectations are and to further explore the extent to which PHI influences public perceptions of the tax‐funded universal healthcare system.

Regarding policy implications, there is definite potential for improvement within the public sector to enhance citizen satisfaction with healthcare services, particularly regarding service quality. By adopting certain practices from the private sector, such as enhanced patient involvement, customer service, better information dissemination, and convenient booking procedures, satisfaction with the public healthcare sector could improve. However, matching the short waiting times and comprehensive nature of services offered by the PHI‐funded sector poses a significant challenge for public healthcare, especially without substantial tax increases.

## 7 Conclusion

This study explored how experiences with PHI influence perceptions of and satisfaction with the public healthcare system. The findings indicate that PHI holders prefer privately funded services primarily due to faster access and better service quality, although the medical quality of specialised hospital care in the public system is still regarded as high.

While PHI offers a sense of security by guaranteeing prompt care, only a small number of respondents expressed willingness to purchase PHI privately if it were not provided by their employer. This suggests that PHI is viewed as a convenience rather than a necessity, and the public healthcare system continues to be seen as broadly satisfactory.

## Data Availability

The data that support the findings of this study are available on request from the corresponding author. The data are not publicly available due to privacy or ethical restrictions.
